# Results of Temporary Stent-assisted Coil Embolization (CATS) for the Treatment of Wide-neck Aneurysms: A 10-year Single Center Experience

**DOI:** 10.1007/s00062-022-01206-6

**Published:** 2022-08-26

**Authors:** F. Gottmann, O. Nikoubashman, A. Höllig, A. Reich, M. Wiesmann

**Affiliations:** 1 Department of Diagnostic and Interventional Neuroradiology, University Hospital RWTH Aachen, Pauwelsstr. 30, 52074 Aachen, Germany; 2 Department of Neurosurgery, University Hospital RWTH Aachen, Aachen, Germany; 3 Department of Neurology, University Hospital RWTH Aachen, Aachen, Germany

**Keywords:** Subarachnoid hemorrhage, Aneurysm treatment, Remodeling, Coil embolization, Recurrance

## Abstract

**Purpose:**

In intracranial wide-neck aneurysms, simple coil embolization is often not a feasible treatment option. Balloon-assisted coiling comes with the drawback of blood flow impairment, whereas permanent stent placement requires long-term antiplatelet therapy. Temporary stent-assisted coiling (coiling assisted by temporary stenting, CATS) is an alternative that eliminates both disadvantages. Because prior studies included only small numbers of patients, it was our aim to analyze the safety and effectiveness of this technique in a larger cohort of patients.

**Methods:**

We retrospectively evaluated all endovascular aneurysm treatments at our institution from 2011 to 2020. Out of a total of 688 aneurysm treatments, we intended to perform 95 (14%) with temporary stent-assisted coiling and included them in our study.

**Results:**

Sixty-four (64)% of aneurysms were acutely ruptured, 3% were symptomatic but unruptured, and 33% were incidental. Successful stent recovery was possible in 93% of treatments. Initial complete and adequate occlusion rate were 53% and 82%, respectively. Long-term follow-up at 6 and 12 months was available for 71% and 44% of cases. Aneurysm recurrence was observed in 10% of cases after 6 months, and in 17% after 1 year or later. Periprocedural complications were noted in 12 cases (13%), of which only 1 complication was definitely associated with temporary stent-assisted coiling (1%). One of the periprocedural complications resulted in neurological damage, the other complications were asymptomatic.

**Conclusion:**

Temporary stent-assisted coiling appears to be a safe and effective treatment method in intracranial wide-neck aneurysms. Procedural safety appears to be comparable with balloon remodeling or permanent stent-assisted coiling, but it comes with the further benefit of diminished need for posttreatment antiplatelet therapy, which may improve the outcome of patients. However, to define the true value and potential benefit of this technique, further prospective studies are required.

## Introduction

Coil embolization is the most established technique for endovascular aneurysm treatment. In wide-neck aneurysms, however, simple coil embolization is often not a feasible option. A variety of adjunct techniques have been introduced to overcome this limitation. The use of balloons helps to safely place coils in the aneurysm. However, blood flow to the brain is impaired during inflation of the balloon, and there is a risk of additional thrombus formation or vessel dissection [1]. Stent-assisted coil embolization does not temporarily interrupt blood flow and helps to stabilize the coils within the aneurysm [2]. However, permanent placement of a stent in an intracranial vessel requires long-term platelet inhibition. Especially in patients with acutely ruptured aneurysms, this may increase complication rates [3].

**Fig. 1:**
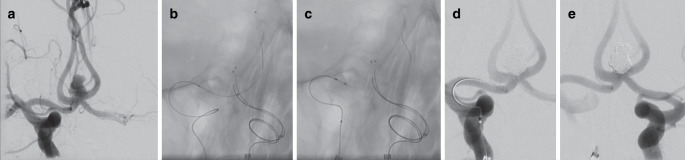
A 55-year-old male patient was admitted for an acute subarachnoid hemorrhage from a wide-necked irregular aneurysm of the anterior communicating artery (AComA) (**a**). To avoid the need of permanent double-antiplatelet therapy during the acute stage of the clinical course it was decided to attempt coil embolization assisted by temporary stenting (CATS). Two Excelsior SL-10 microcatheters (Stryker Neurovascular, Fremont, CA, USA) were advanced into the aneurysm through the A1 segment of the left anterior cerebral artery (ACA). Then a third Excelsior SL-10 microcatheter was advanced through the A1 segment of the right ACA and the AComA into the A2 segment of the left ACA (**b**). Next, a self-expanding braided stent (LEO 2.5 × 18 mm, Balt, Montmorency, France) was introduced into the third microcatheter and partially expanded by pulling back the microcatheter (**c**). Thus, the aneurysm neck was covered by the stent. The aneurysm was completely embolized using 15 bioactive Micrus Cerecyte coils (Codman Neurovascular, Raynham, MA, USA) (**d**). By resheathing the stent it was verified that the coils were stable, and all microcatheters were removed (**e**)

To overcome this limitation, temporary stent-assisted coil embolization (coiling assisted by temporary stenting, CATS) has been proposed as an alternative method, with the advantage that blood flow is not interrupted during treatment and no implants are left behind in the parent vessel [4]. Two microcatheters are needed for this technique: The first microcatheter is advanced into the aneurysm to perform coil embolization. The second microcatheter is used to deploy a stent or detachable stent-retriever and hereby cover the aneurysm neck without permanently releasing the stent. The microcatheter can be placed in the aneurysm either before or after stent deployment [5]. After completion of coil embolization, the stent is recovered (Fig. 1). If there is coil protrusion during stent recovery, the stent can be redeployed and released for permanent implantation. Variations of CATS using a third microcatheter are possible, such as the for double microcatheter embolization (Fig. 1), for additional microcatheter remodeling, or for double temporary stent remodeling.

The CATS is an established standard technique in our institution for the treatment of wide-neck aneurysms. To date, only case reports and small case series on this technique have been published [4, 6–10]. To assess safety and effectiveness of CATS, we performed a retrospective intention-to-treat analysis of all consecutive patients, who were intended to be treated with CATS over a period of 10 years.

## Material and Methods

### Patients

After approval from our local ethics committee (EK055/21), we retrospectively searched our clinical database for all patients who either received CATS or were intended to be treated with CATS in our institution between January 2011 and December 2020. During this period, a total number of 688 endovascular treatments of incidental and acutely ruptured aneurysms were performed. It is routine practice in our institution that aneurysm treatment decisions are made in a joint discussion between neurologists, neurosurgeons, and neuroradiologists, and to document the planned treatment modality. We identified and included all consecutive 95/688 (14%) aneurysm treatments, which were performed with CATS or were intended to be performed with CATS. Therefore, this analysis also includes patients, in whom endovascular treatment was modified or not feasible at all.

### Clinical and Procedural Data

Baseline characteristics were, among others, location and maximum diameter of the aneurysm, the neck-to-dome ratio, and the diameter of the parent vessel. Indication for treatment, materials used, and periinterventional and posttreatment platelet inhibition management were recorded for all patients.

Primary outcome parameters were degree of occlusion according to the modified Raymond-Roy classification after coiling as well as 6 and 12 months after treatment [11]. This classification discriminates between complete occlusion of the aneurysm (modified Raymond and Roy class I), residual filling of the aneurysm neck (class II), contrast filling within coil interstices (class IIIa), and residual perfusion at the neck and side (class IIIb). The term “adequate occlusion” was used for aneurysms which were either completely occluded or in which only residual filling of the aneurysm neck was noted (class I or II) [12]. Secondary outcome parameters were procedure-related complication rates (including stent recovery rates) and clinical outcome using the modified Rankin scale (mRS) after 12 months [13].

### Statistical Analysis

Data distribution was tested with a Shapiro-Wilk test. Non-parametric data are indicated as median with interquartile range (IQR), whereas parametric data are indicated as mean with standard deviation (SD). Correlations between variables were calculated by using the Pearson correlation coefficients. Differences between groups were examined using the Mann-Whitney‑U test. *P* values < 0.05 were considered significant. An additional Holm-Bonferroni correction was performed in cases of multiple testing if first level results were found to be significant. All statistical analyses were performed with SPSS 28 software (IBM, Armonk, NY, USA). The number of patients lost to follow-up is reported.

We used the STROBE cohort checklist when writing our report [14]. This study was approved by the appropriate ethics committee and has been performed in accordance with the ethical standards laid down in the 1964 Declaration of Helsinki and its later amendments.

## Results

### Baseline Characteristics

The 95 aneurysm treatments took place in 87 patients (60 female, 69%) with 1–2 treated aneurysms per patient. Mean age of patients was 58.1 ± 11.7 years, ranging from 29 to 84 years. Of all 95 aneurysms, 61 (64%) were acutely ruptured, 3 (3%) were symptomatic but unruptured, and 31 (33%) were incidental.

Overall, 33 (35%) aneurysms were located at the anterior communicating artery; 27 (28%) at the internal carotid artery; 12 (13%) at the basilar artery (with 8 of these located at the top of the basilar artery); 11 (12%) at the A1 segment of the anterior cerebral artery (with 6 of these located close to the carotid-T); 4 (4%) at the middle cerebral artery; 3 (3%) at the anterior choroidal artery; 2 (2%) at the pericallosal artery; 1 (1%) at the vertebral artery; 1 (1%) at the posterior communicating artery and 1 (1%) at the posterior inferior cerebellar artery.

The average maximum aneurysm diameter was 6.8 ± 3.4 mm, ranging from 1.9 to 19.6 mm. Average neck diameter was 3.6 ± 1.5 mm, ranging from 1.3 to 9.2 mm. Average dome diameter was 5.5 ± 3.5 mm, ranging from 1.3 to 19.6 mm. Average neck-to-dome ratio was 0.7 ± 0.3, ranging from 0.2 to 1.5. Average parent vessel diameters were 2.5 ± 0.8 mm, ranging from 0.9 to 4.5 mm.

### Procedural Characteristics

A Solitaire AB stent (Medtronic, Irvine, CA, USA) was used in 87/95 (92%) aneurysms, a Barrel stent (Medtronic) was used in 4 (4%) aneurysms, an Enterprise stent (Codman Neurovascular, Raynham, MA, USA) was used in 3 (3%) aneurysms, and a LEO stent (Balt, Montmorency, France) was used in 1 (1%) aneurysm. A stent was implanted permanently in 7 of 95 (7%) treatments.

In 3 of 95 procedures the stent was expanded over the aneurysm neck first, and the microcatheter for coil embolization was entered into the aneurysm through the mesh of the stent. In 92 of 95 procedures the microcatheter was placed in the aneurysm first before the stent was expanded (jailing technique). In 13 of these 92 procedures the interventionalist later decided to pull the microcatheter back and navigate through the mesh of the stent to a different compartment of the aneurysm for placement of additional coils. This was successful in 12 of 13 cases. In one case this proved difficult and the interventionalist decided against further attempts since acceptable coil embolization had already been achieved.

In 87 of 95 procedures the standard technique of CATS with 2 microcatheters was used. In 8 cases 3 microcatheters were used for double microcatheter embolization (*n* = 3), additional microcatheter remodeling of a branching vessel (*n* = 4), or double temporary stent remodeling (*n* = 1).

Ninety-two of 95 (97%) procedures were performed with platelet inhibition during the procedure, with 88 patients receiving intravenous (IV) tirofiban, 2 patients receiving IV ASA, and 2 patients being pretreated with dual platelet inhibition (ASA and clopidogrel). In 3 cases, the procedure was performed without platelet inhibition.

After the procedure, there was no further platelet inhibition after discharge in 47 cases; in 13 cases, ASA was prescribed for up to 2 weeks; in 27 cases, ASA was prescribed for a period between 4 weeks and 6 months; in 1 case, ASA was prescribed indefinitely; in 10 of the cases with prolonged ASA treatment, there was dual platelet inhibition with additional clopidogrel for a period of 4 weeks to 6 months. In the 7 patients, in whom the stent was implanted permanently, 6 received dual platelet inhibition for at least 3 months and indefinite ASA treatment. The remaining patient died during hospitalization.

### Primary Outcome

Initial treatment success: 50 (53%) aneurysms were completely occluded (modified Raymond and Roy class I), 28 (29%) aneurysms had a residual neck (class II), 8 (8%) aneurysms had contrast filling within coil interstices (class IIIa), and 9 (9%) aneurysms showed residual perfusion at the neck and side (dog-ear) (class IIIb). Hence, adequate occlusion including aneurysms with complete occlusion and residual neck (classes I and II) was achieved in 82% (78/95). There was no significant correlation between initial occlusion rate and maximum aneurysm diameter (*p* = 0.73), aneurysm neck diameter (*p* = 0.44), dome-to-neck ratio (*p* = 0.69), and parent artery diameter (*p* = 0.40).

Follow-up at 6 months: follow-up imaging with magnetic resonance (MR) time-of-flight (TOF) angiography and/or digital subtraction angiography (DSA) after 6 months was available for 67/95 (71%) aneurysms. At 6 months, there was recurrence in 7/67 (10%) aneurysms, whereas the remaining 60 (90%) were stable or improving. In 1 of these 7 cases no need for secondary treatment was seen. In the other 6 cases additional endovascular procedures were performed to treat the aneurysm. There was a significant correlation between aneurysm neck diameter and the risk of recurrence at 6 months (*p* = 0.01). Risk of recurrence was not associated with initial aneurysm occlusion grade (*p* = 0.59). There was no significant correlation between recurrence and maximum aneurysm diameter (*p* = 0.37), neck-to-dome ratio (*p* = 0.57), and parent artery diameter (*p* = 0.94).

Follow-up at 12 months: follow-up imaging with MR TOF angiography and/or DSA after 12 months was available for 42/95 (44%) aneurysms. At 12 months, there was recurrence in 7/42 (17%) aneurysms (1 additional compared to after 6 months), whereas the remaining 35 (83%) were stable or improving. In 2 of these 7 cases no need for secondary treatment was seen. In the other 5 cases additional endovascular procedures were performed to treat the aneurysm. There was a significant correlation between aneurysm neck diameter and the risk of recurrence at 12 months (*p* = 0.04). Risk of recurrence was not associated with initial aneurysm occlusion grade (*p* = 0.41). There was no significant correlation between recurrence and maximum aneurysm diameter (*p* = 0.67), neck-to-dome ratio (*p* = 0.51), and parent artery diameter (*p* = 0.20).

### Secondary Outcome

In 7/95 (7%) aneurysms, it was not possible to safely recover the stent due to impending coil migration into the parent artery. When retrieval of the stent was slowly initiated, the interventionalist observed some degree of outward movement of the coils and decided to fully reopen the stent. Hence, the overall success rate of stent recovery was 93%. These 7 treatments were thus finished as permanent stent-assisted coil embolization.

However, there were no instances of unsuccessful attempted stent retrieval due to a technical inability to retrieve the stent, particularly for those stents that are not electrolytically detached. Thus, the technical success rate of stent recovery was 100%.

There was a significant correlation between success of stent recovery and neck-to-dome ratio (*p* = 0.03) with larger neck-to-dome ratios favoring successful stent recovery. However, this result was not significant after correction for multiple testing. There was no significant correlation between success of stent-recovery and maximum aneurysm diameter (*p* = 0.76), aneurysm neck diameter (*p* = 0.30), and parent artery diameter (*p* = 0.98).

Periprocedural complications occurred in 12/95 (12.6%) procedures:

Periinterventional bleeding from the aneurysm occurred in 3 cases: In all cases embolization was started under IV tirofiban treatment. Bleeding was caused by perforation of the aneurysm during placement of the first, second, and third coil, respectively. In all cases, the bleeding ceased quickly when coil embolization was continued. Precisely, bleeding ceased when the perforating coil was fully placed, or after placing one or two additional coils, respectively. Notably, all three periinterventional bleedings occurred in acutely ruptured aneurysms. On the other hand, no periinterventional bleedings occurred in elective cases. This corresponds to a periinterventional bleeding rate of 4.9% in acutely ruptured aneurysms and 0% in elective cases. To the best of our knowledge, no additional morbidity was caused by the three periinterventional bleedings.

Thromboembolic complications occurred in 3 cases: in two cases, small peripheral thromboembolic vessel occlusions were noted during angiography. In one of these two cases, a small infarction in the parietal region of the middle cerebral artery territory was observed. In the second of these two cases no territorial infarction was found on MR imaging after 24 h. In the remaining third case, peripheral thromboembolic infarctions were observed on follow-up MR imaging. However, this case was inconclusive because the patient suffered from bihemispheric lesions and atrial fibrillation, which may as well have been the source of infarction. All of these three cases were performed with the patient under periinterventional platelet inhibition with IV tirofiban. No specific morbidity could be attributed to the three thromboembolic complications.

Local thrombus formation occurred in 4 cases:

In one case, during embolization of a hemorrhagic giant aneurysm of the internal carotid artery under IV tirofiban treatment an occlusion of the stent was observed. This happened when the aneurysm was already almost completely filled with coils but the interventionalist had decided to reinsert the microcatheter to reach a different part of the aneurysm. Probably, thrombus which had formed between coil loops was pushed back into the parent vessel during this maneuver. The thrombus was successfully removed by partial closure and retrieval of the stent but at this point subarachnoid hemorrhage from the aneurysm neck was noted. The most probable explanation for this was that the microwire, which had been used to reinsert the microcatheter into the aneurysm, had perforated the vessel wall unnoticed. Lastly, it was necessary to occlude the internal carotid artery through coil embolization to stop the hemorrhage. There was cross flow from the contralateral hemisphere but the patient suffered a partial middle cerebral artery infarction resulting in left-sided hemiparesis.

In another case, there was thrombus formation from the aneurysm neck into the parent artery after removal of the microcatheters; the thrombus was dissolved by intra-arterial administration of 45 mg rtPA. No peripheral infarction or resulting morbidity was observed. In another case, there was thrombus formation in the parent artery after removing a stretched coil, which was dissolved by intra-arterial administration of 42 mg of rtPA. No peripheral infarction or resulting morbidity was observed. In the last case, thrombus formation in the parent artery was observed after removal of the microcatheters. Since the thrombus led to occlusion of the artery, thrombectomy was performed successfully using a stent-retriever. During this maneuver one of the coils in the aneurysm became entangled with the stent-retriever but could be removed together with the thrombus without further problems. No peripheral infarction or resulting morbidity was observed. All of these four cases were performed with the patient under periinterventional platelet inhibition with IV tirofiban.

Notably, two of the three thromboembolic complications and all four cases of local thrombus formation occurred in acutely ruptured aneurysms, whereas in elective cases only one thromboembolic complication was observed.

There was displacement of a coil during removal of the stent in one case of an acutely ruptured aneurysm. The coil was removed without complications using the temporary stent (Solitaire AB) for extraction as described previously [15].

A minor dissection of the cervical part of the internal carotid artery without need for specific treatment and without associated morbidity occurred in 1 case of an acutely ruptured aneurysm.

In total, 11 of the 12 complications occurred in acutely ruptured aneurysms. Only 1 complication occurred in the elective cases. Only 1 of the 12 complications resulted in neurological damage, to the best of our knowledge the other 11 complications were asymptomatic.

Statistical analysis showed that the complication rate was significantly higher in acutely ruptured cases than in elective cases (*p* = 0.02). However, this result was not significant after correction for multiple testing. There was no significant correlation between the risk of periprocedural complication and aneurysm diameter (*p* = 0.27), aneurysm neck diameter (*p* = 0.51), neck-to-dome ratio (*p* = 0.25), and parent artery diameter (*p* = 0.51).

Neurological outcome at discharge was as follows: mRS = 0, *n* = 42; mRS = 1, *n* = 12; mRS = 2, *n* = 8; mRS = 3, *n* = 8; mRS = 4, *n* = 4; mRS = 5, *n* = 7; mRS = 6, *n* = 16. Thus, in our cohort at discharge temporary neurological morbidity (mRS = 3–5) was 19/97 (19.6%), and mortality was 16/97 (16.5%). Neurological outcome at 6 months was as follows: mRS = 0, *n* = 48; mRS = 1, *n* = 11; mRS = 2, *n* = 7; mRS = 3, *n* = 8; mRS = 4, *n* = 4; mRS = 5, *n* = 3; mRS = 6, *n* = 16. Thus, in our cohort at 6 months neurological morbidity (mRS = 3–5) was 15/97 (15.5%), and mortality was 16/97 (16.5%).

## Discussion

Temporary stenting has been suggested as an alternative to the well-established remodeling techniques of balloon-assisted coil embolization and stent-assisted coil embolization. To the best of our knowledge, however, since the first report on this technique in 2013, only 6 case reports or small case series have been published with a total number of reported cases of 65 [4, 6–10]. This already included 33 cases from our own group. We thus performed a retrospective intention-to-treat analysis on 97 aneurysm treatments to expand on this and determine the safety and effectiveness of this technique.

The results of our study validate the technique of using a temporary stent to assist coil embolization of wide-neck aneurysms as practical and effective. The stents could be successfully retrieved in 93% of cases. In the remaining cases stent recovery was deemed unsafe due to impending coil migration into the parent artery, and the treatment was finished as permanent stent-assisted coil embolization without complications. Thus, the success rate of CATS in terms of whether it can avoid a permanent stent implantation in wide-necked aneurysms is 93%. However, there were no instances of unsuccessful attempted stent retrieval due to a technical inability to retrieve the stent in cases when interventionalists had considered it safe to try removing the stent. Thus, the technical success rate of stent recovery was 100%.

In our cohort, we achieved an adequate aneurysm occlusion, including a neck remnant (Raymond-Roy classes I + II), in 82% of cases. Looking at immediate effectiveness of treatment, our results appear to be comparable with previous studies, indicating stent-assisted coiling to be superior to balloon remodeling regarding aneurysm occlusion [16–18].

Our study is the first to report follow-up data on patients treated with temporary stent-assisted coil embolization. With recurrence rates of 10% after 6 months and 17% after 1 year or later, our results appear both acceptable and comparable with results achieved using permanent stent-placement or balloon remodeling [16–18]. It has been reported that initial aneurysm occlusion as characterized by the Raymond-Roy classification is a predictor for recurrence [19]. In our cohort, we did not find such a correlation between the grade of initial aneurysm occlusion and the risk of recurrences at both 6 and 12 months. However, this may be due to the limited size of our sample. In our patients the diameter of the aneurysm neck was the only significant predictor of aneurysm recurrence. The validity of our findings is, however, impaired by the suboptimal follow-up rates of our cohort, which were caused by the retrospective design of our study, as well as partly by the COVID pandemic.

We observed a complication rate of 12.6%. This appears to be comparable to the techniques of balloon remodeling and permanent stent-assisted coiling, for which also complication rates between 10% and 20% have been reported [20–22]. However, we would like to draw attention to the fact that it was our intention to record all kinds of unintended events during the procedure as complications, even when they were not associated with the procedure of temporary stenting (e.g., stretching of coils). We did this to allow a thorough evaluation of our technique. Therefore, it should be noted that our complication rate of 12.6% does not correspond to the specific risks of temporary stenting. While several events were not clearly attributable to our technique (e.g., thrombus formation), we also observed an event of coil displacement during stent removal, which is definitely a complication associated with temporary stenting. Therefore, at least one of our complications was clearly associated with temporary stenting (1%). However, from a scientific perspective, we cannot state a valid complication rate specifically related to the use of temporary stenting. Still, it is safe to assume that this measure was considerably smaller than 12.6% in our study.

With regard to complication rates it also needs to be considered that our study is a real-world best-practice report. This means that we also included interventions performed by younger, less experienced interventionalists, even when it was the first time they used this method. Then, it needs to be considered that the majority of treated aneurysms in our study were acutely ruptured. It is reasonable to expect that complication rates in acutely ruptured cases will be higher than in elective cases. For one thing, acutely ruptured aneurysms carry a higher risk for periinterventional rupture than unruptured aneurysms. Consequently, all three periprocedural aneurysm ruptures occurred in acutely ruptured cases, whereas our periprocedural aneurysm rupture rate in elective cases was zero. In addition, there is evidence that the coagulation system is being activated following acute SAH. Although the evidence regarding this is not entirely clear, it has been speculated whether the risk for thrombus formation may be higher in acutely ruptured cases. Consequently, only one case of thromboembolism occurred in one of our elective cases. The two remaining complications of thromboembolism and all four cases of local thrombus formation occurred in acutely ruptured aneurysms. As expected, complications occurred significantly more frequent in acutely ruptured cases than in elective cases. In total, 11 of the 12 complications occurred in acutely ruptured cases, whereas only one complication occurred in the elective cases.

Although the majority of our aneurysms were acutely ruptured, the vast majority of treatments were performed with platelet inhibition during the procedure, with 88 patients receiving intravenous tirofiban, and 2 patients being pretreated with dual platelet inhibition. This strategy is in accordance with Almekhlafi and Goyal who have also recommended effective platelet inhibition during temporary stent-assisted coil embolization to prevent thromboembolic complications [23]. As compared to permanent stent placement, the majority of patients in our cohort did not require dual-antiplatelet therapy following embolization. This may be considered an advantage, since it has been shown that dual-antiplatelet therapy increases the risk of occurrence of hemorrhagic events after aneurysm embolization [3, 24]. Temporary stenting may thus be especially useful for patients with an acutely ruptured aneurysm. The nature of our study, however, does neither allow to confirm this hypothesis nor to determine the size of this effect in our cohort.

There are different opinions between interventionalists whether the microcatheter for coil embolization should be placed in the aneurysm first before the stent is expanded (jailing technique), or whether the microcatheter should be navigated into the aneurysm through the mesh of the expanded stent. In our institution usually the jailing technique is being used. However, our results show that the microcatheter can be pulled back and navigated through the mesh of the stent should this prove to be necessary during embolization.

Recently, the Comaneci device has been introduced. This stent-like device also allows temporary remodeling during coil embolization of aneurysms. Sirakov et al. reported the use of this device in 118 acutely ruptured aneurysms with rates for technical success and aneurysm recurrence comparable to our technique, and a device-related complication rate of 10% [25]. A drawback of this technique, however, is that the device cannot be detached if coil protrusion is observed at the end of the procedure. Lately, the Comaneci device has also been used in our institution in a few cases with good results. It seems to us, that while use of the Comaneci device and the technique of CATS as described in this paper are in essence quite similar, there are also some differences: The expansion of the Comaneci device can to some extent be controlled by the user. This may allow for better adaptation to the aneurysm neck in some cases, whereas on the other hand it could increase complication rates following too forceful expansion. On the other hand, when the interventionalist starts to close the remodeling device at the end of the procedure and realizes that the coil package is not stable, CATS has an advantage over the Comaneci device. In a CATS procedure, the stent can be instantly redeployed, thus preventing full protrusion of the coils into the parent vessel. The Comaneci device needs to be completely removed, and a new catheter be placed before deployment of a permanent stent. Admittedly, only in a small number of cases will this potentially make a difference in interventional outcome.

In the past years alternatives to coil embolization for wide-necked bifurcation aneurysms have been introduced, such as the WovenEndobridge (WEB) device, the Contour Neurovascular System, or Flow diverters. These techniques may be an alternative to treat ruptured aneurysms with low morbidity and mortality [22, 26, 27]. However, prospective randomized studies to compare the various treatment methods have yet to be performed.

In conclusion, our data show that temporary stent-assisted coil embolization may be a safe and effective alternative to other remodeling techniques. In comparison to permanent stent placement, patients do not require long-term platelet inhibition. In comparison to balloon remodeling, it has the benefit of avoiding temporary blood flow inhibition. Potentially, a disadvantage of CATS may be that there is the risk of coil loops intermingling with the stent. However, this risk appears to be rather small since we did not observe such a complication in our cohort. In addition, especially for younger interventionalists CATS may be considered a technically easier approach than balloon remodeling [6, 8].

However, although the literature on the results of the various strategies for aneurysm embolization is extensive, there is no prospective randomized study to compare the different methods with their respective advantages and disadvantages against each other.

## Limitations

The limitations of our study are a possible selection bias as the patients in our study were not randomized beforehand to the opposing treatment techniques for wide-neck aneurysms. To reduce this possible effect, however, we conducted our study as an intention-to-treat analysis. A second limitation of our study is that we were able to collect a 6-month FU in 71% of patients, and a 12-month FU in 44% of patients only. This is due to the retrospective design of our study and the fact that it covers a period of more than 10 years. Prospective studies with long-term follow-up data are required to compare embolization methods. Other limitations are that antiplatelet medication protocols varied within our cohort, and that our results were not evaluated by an independent core laboratory. To minimize this potential bias, we did not rely on the interventional reports but re-analyzed all images. Moreover, there is some potential inhomogeneity due to the use of three different stents.

## Conclusion

In this retrospective intention-to-treat study, temporary stenting appeared to be an effective technique for the treatment of wide-neck aneurysms. Procedural safety appeared to be comparable to balloon remodeling and permanent stent-assisted coiling. The major advantage of our treatment may be a diminished need for posttreatment antiplatelet therapy, which might cause cerebral hemorrhage or complicate further treatment in the intensive care unit. We thus consider it possible that this technique may be particularly useful for patients with an acutely ruptured intracranial aneurysm. However, to define the true value and potential benefit of this technique, further prospective studies comparing it to other techniques for treatment of wide-necked aneurysms are required.
